# Photodynamic Therapy in Orthodontics: A Literature Review

**DOI:** 10.3390/pharmaceutics13050720

**Published:** 2021-05-14

**Authors:** Marcin Olek, Agnieszka Machorowska-Pieniążek, Wojciech Stós, Janusz Kalukin, Dorota Bartusik-Aebisher, David Aebisher, Grzegorz Cieślar, Aleksandra Kawczyk-Krupka

**Affiliations:** 1 Department of Orthodontics, Faculty of Medical Sciences in Zabrze, Medical University of Silesia, 40-055 Katowice, Poland; d200922@365.sum.edu.pl (M.O.); agamach@onet.pl (A.M.-P.); 2 Department of Orthodontics, Dental Institute, Faculty of Medicine, Jagiellonian University Medical College, 31-155 Cracow, Poland; wojciech.stos@uj.edu.pl (W.S.); janusz.kalukin@uj.edu.pl (J.K.); 3 Department of Biochemistry and General Chemistry, Faculty of Medicine, University of Rzeszów, Kopisto 2A, 35-310 Rzeszów, Poland; dbartusik-aebisher@ur.edu.pl; 4 Department of Photomedicine and Physical Chemistry, Faculty of Medicine, University of Rzeszów, Kopisto 2A, 35-310 Rzeszów, Poland; daebisher@ur.edu.pl; 5 Department of Internal Medicine, Angiology and Physical Medicine, Center for Laser Diagnostics and Therapy, Faculty of Medical Sciences in Zabrze, Medical University of Silesia, 40-055 Katowice, Poland; cieslar1@tlen.pl

**Keywords:** photodynamic therapy, orthodontics, photosensitizer(s)

## Abstract

Treatment of malocclusions using fixed orthodontic appliances makes it difficult for patients to perform hygiene procedures. Insufficient removal of bacterial biofilm can cause enamel demineralization, manifesting by visible white spot lesions or periodontal diseases, such as gingivitis periodontitis or gingival hyperplasia. The classic methods of preventing the above problems include, in addition to proper hygiene, ultrasonic scaling, periodontal debridement, and oral rinses based on chlorhexidine. New alternative methods of reducing plaque around brackets are being developed. There is a growing interest among researchers in the possibility of using photodynamic therapy in orthodontics. A literature search for articles corresponding to the topic of this review was performed using the PubMed and Scopus databases and the following keywords: ‘photodynamic therapy’, ‘orthodontics’, and ‘photosensitizer(s)’. Based on the literature review, two main directions of research can be distinguished: clinical research on the use of photodynamic therapy in the prevention of white spot lesions and periodontal diseases, and ex vivo research using a modified orthodontic adhesive by adding photosensitizers to them. Methylene blue is the most frequently used photosensitizer in clinical trials. The effectiveness of antimicrobial photodynamic therapy is mainly compared to the ultrasonic scaler as a single therapy or as an adjunct to the ultrasonic scaler. In their conclusions, the researchers most often emphasize the effectiveness of antimicrobial photodynamic therapy in reducing microbial levels in patients treated with fixed appliances and the possibility of using it as an alternative to routine procedures aimed at maintaining a healthy periodontium. The authors suggest further research on the use of photodynamic therapy to prove the validity of this method in orthodontics. It should also not be forgotten that proper hygiene is the basis for maintaining oral cavity health, and its neglect is a contraindication to orthodontic treatment.

## 1. Introduction

**Figure 1:**
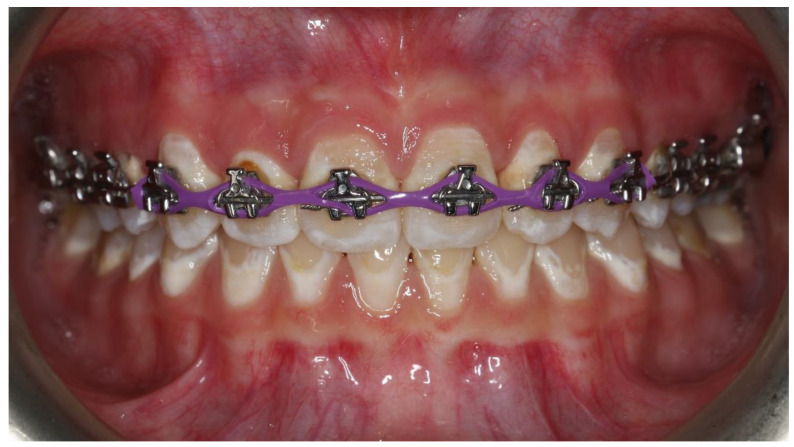
White spot lesions caused by insufficient oral hygiene during orthodontic treatment.

Treatment of malocclusions using fixed orthodontic appliances makes it difficult for patients to perform hygiene procedures. Brackets bonded to the teeth create new places of retention for food debris and resident bacterial microflora, creating a biofilm in the form of plaque [1,2]. In cases of insufficient compliance with the hygiene regime, the persistent plaque and the products of bacterial metabolism content cause enamel demineralization, which may eventually progress to the formation of a carious cavity. Enamel demineralization is manifest by visible white spot lesions (WSL) around the brackets (Figure 1). The already known methods of preventing the formation of WSL include, among others, appropriately motivating the patient and instructing oral hygiene and the use of sealants or fluoride varnishes [2,3,4,5].

**Figure 2:**
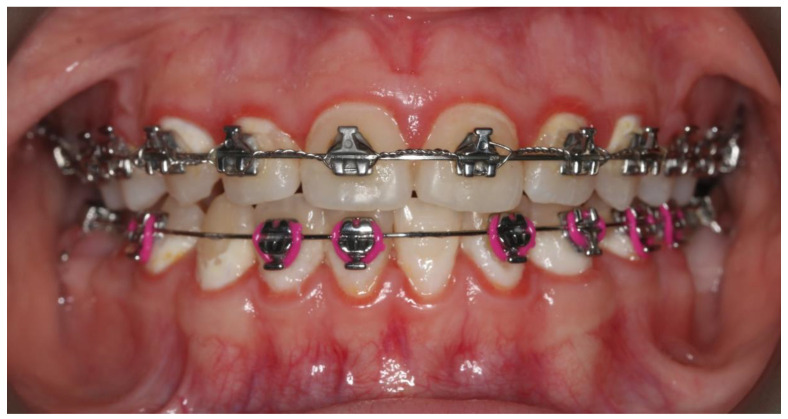
Gingivitis associated with dental plaque.

Insufficient removal of the bacterial biofilm can also cause periodontal diseases, such as gingivitis (Figure 2), periodontitis, recession, or gingival hyperplasia [1,5,6,7]. The classic methods of preventing the above diseases include, in addition to proper hygiene, ultrasonic scaling (US), periodontal debridement (PD), and oral rinses based on chlorhexidine. However, new alternative methods of reducing plaque around brackets are developing.

From the clinician’s perspective, the desired factor is the acceleration of teeth movement without side effects, such as root resorption. Corticotomy is a more widely described method for this purpose, but it is an invasive procedure [8,9]. The use of lasers in this field in the form of biomodulation is known, making it possible to accelerate the movement of teeth and shorten the treatment time [10,11].

A relatively modern therapeutic method is photodynamic therapy (PDT). It is successfully used in neoplastic and precancerous lesions of the gastrointestinal tract [12,13,14], as well as in dermatological [15] and urological diseases [16]. PDT is more often used in oral diseases such as lichen planus [17] or oral squamous cell carcinoma [18,19,20]. Antimicrobial use antimicrobial photodynamic therapy (aPDT) has the advantage of having broad-spectrum activity, including gram-negative bacteria, gram-positive bacteria, yeast, and fungi, with no resistance being produced [21]. Unsurprisingly, the use of aPDT in dentistry is growing in popularity, mainly in periodontal and mucosal diseases, but also for alternative purposes such as accelerating the movement of the teeth.

Three components are necessary for the photodynamic action, namely photosensitizer, light, and oxygen. Photosensitizers (PSs) are natural or synthetic compounds, each with a unique wavelength needed for activation [22]. Excited PS reacts with oxygen to form reactive oxygen species (ROS), and the reaction destroys bacterial cells [23]. Healthy tissues may remain intact if the PS, wavelength, light energy, and drug-light interval are correctly selected [22,23].

## 2. Materials and Methods

**Figure 3:**
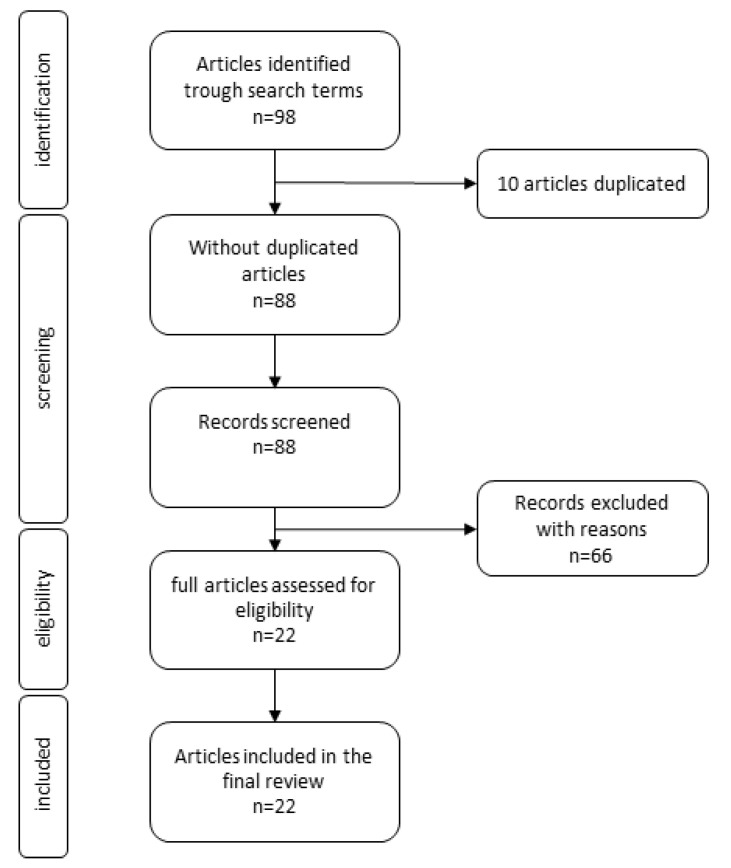
PRISMA flow diagram showing the study selection and identification.

“PubMed” and “Scopus” were searched from inception to March 2021 for studies on the use of PDT in orthodontics. The search was carried out using the keywords “photodynamic therapy” and “orthodontics”. The present systematic review was reported based on guidelines of Preferred Reporting Items for Systematic Reviews and Meta-Analysis (PRISMA) Statement (Figure 3) and Cochrane Collaboration recommendations [24]. Both clinical trials (Table 1) and ex vivo studies (Table 2) were selected for the literature review, which is further divided into two chapters. After reading the abstracts and full texts of articles, the papers consistent with the review subject were selected. Studies without access to the full article or in a language other than English were disqualified. In addition, reviews, case reports, comments, and letters to the editor were excluded.

## 3. Clinical Trials

### 3.1. Comparison of Ultrasonic Scaling and Antimicrobial Photodynamic Therapy

Abellan et al. (2019) conducted studies about the effectiveness of PDT and US on periodontal health in patients treated with fixed braces. They divided 20 people aged 12 to 18 into two groups who underwent seven sessions (days 0, 15, 30, 45, 90, 180, 270) of PDT or US. The researchers showed a statistically significant decrease in the plaque index (PI), gingival index (GI), and probing depth (PD) in both groups compared to the starting point, but no differences between PDT and US. The multiplex map of human high sensitivity immunoassays showed a decrease in IL-1b, IL-10, TNF-a levels, persistent levels of IL-6, IL-1ra, and an increase in FGF-2, but no differences for both groups. In both groups, there was a similar decrease in colony-forming units (CFU) for *P. gingivalis*, *P. intermedia*, and *F. nucleatum*. The researchers emphasize that PDT can be considered a safe alternative to gingivitis therapy with a slightly longer-lasting effect [25].

Gomez et al. (2018) assessed the effectiveness of US and PDT in preventing gingivitis and white spot lesions during orthodontic treatment. Patients received seven PDT and US sessions, four sessions at two-week intervals at baseline (T0), followed by three boosters every three months (T1, T2, T3). In the international caries detection and assessment system (ICDAS), starting from T1, a slight increase was observed. However, these differences were not statistically significant. Periodontal indicators reduced their levels. full-mouth bleeding score (FMBS) and PD recorded the most significant decrease in T1 and full-mouth plaque score (FMPS) in T2, with no differences between the PDT and the US group. In the studies on a microbiome, the number of periopathogens and cariogenic bacteria decreased, without statistically significant variations between the two groups [26].

### 3.2. Comparison of Ultrasonic Scaling and Ultrasonic Scaling with Adjunct Antimicrobial Photodynamic Therapy

Al Nazeh et al. (2020) divided the 22 subjects into two groups. The first group was subject to US only and the second group to both US and PDT. Methylene blue at a concentration of 0.0005% was used as a photosensitizer and then irradiated with light at a wavelength of 670 nm, fluence 22 J/cm^2^, and fluence rate 150 mW. Before the intervention, one week after, and four weeks after, the following clinical parameters were assessed: dichotomous recording for plaque scores (PS) and bleeding on probing (BOP). The researchers collected plaque samples for bacterial analysis. There was a statistically significant decrease in PS and BoP for both groups, compared to the baseline values, but no difference between them. In the US group, there was a decrease in CFU for *T. forsythia* after one week. In turn, for the US-and-PDT group, there was a statistically significant decrease in CFU for *T. forsythia* and *P. gingivalis* both after one week and four weeks [27].

Alqerban (2020) compared the effectiveness of US with adjunct aPDT and US with adjunct photobiomodulation (PBM) to US alone in anti-gingivitis therapy in patients treated with fixed appliances. The researcher divided 45 patients into three equal groups. Alqerban measured PD, BoP, and PS in each group and collected plaque samples and gingival crevicular fluid (GCF) at baseline and on days 30 and 60 of the study. In the group with aPDT, methylene blue with a concentration of 0.0005% was used as a photosensitizer and then irradiated with light with the following parameters: 670 nm, 22 J/cm^2^, 150 mW. There was a statistically significant decrease in PS and BoP in all groups compared to the baseline, with no variation between each group. In the aPDT group, there was a progressive decrease in PD, yet the variations between the groups were not statistically significant. The level of Human β-defensins in GCF assessed through the ELISA test showed a statistically significant decrease after 30 days for all groups. In the aPDT group, there was a further decrease observed after 60 days and a decrease in *T. denticola* after 30 and 60 days [28].

Alshahrani et al. (2020) compared the use of full- mouth periodontal debridement (FMPD) and FMPD with adjunct PDT. Yet another PS was MB in a concentration of 0.0005%, which they photoactivated with light with the following properties: 670 nm, 22 J/cm^2^, 150 mW. A total of 26 people diagnosed with an orthodontic treatment-induced gingival enlargement (OTGE) were qualified for the study and divided into two research groups. In the clinical examination, the researchers assessed the values of PS, BOP, PD, and hyperplastic index (HI) at baseline, two and four weeks after the applied therapy. There was a statistically significant decrease in value for all items in both groups after two and four weeks. It is worth noting that there was a statistically significant decrease in HI for the FMPD-PDT group compared to the FMPD group in both time intervals. There was a significant reduction in *P. gingivalis*, *T. forsythia*, and *T. denticola* from baseline. A statistically significantly higher reduction in mean log CFU/mL for the FMPD-PDT group after two and four weeks was observed for *P. gingivalis*, *T. forsythia*, while for *T. denticola*, a significant difference occurred only after a two-week follow-up. In ELISA, IL-1β showed a significant reduction after four weeks of observation for both groups. There were significant differences in the reduced levels of IL-6 between the groups, to the FMPD-PDT group’s benefit, after a four-week follow up [29].

Baeshen et al. (2020) assessed the effect of PDT on clinical parameters, pain levels, cytokine secretion, and bacterial microflora in adolescent orthodontic patients with gingivitis. Group 1 underwent US and PDT treatment using MB as a PS, while Group 2 underwent US only. However, when comparing the groups with each other, there were no differences in PS and BoP. There was a statistically significant decrease in the value in favor of the group with adjunct PDT. The use of the therapies did not change the level of perceived pain and PD values. There was a significant decrease in *T. forsythia* for Group 1 compared to Group 2, but only after a one-week follow up. After four weeks, there was a re-increase in microbial counts. The researchers demonstrated a significant decrease in TNF-α and IL-6 for both groups compared to baseline. For TNF-α, a significant difference between the groups was present at week four and for the second cytokine at week one [30].

Kamran (2020) also compared US alone with US with adjunct PDT. The latter used MB as a PS. The studies showed a reduction in PS and BoP compared to baseline for both groups after three weeks and further after six weeks. In both groups, the PS and BoP’s value reduced significantly after six and three and six weeks, respectively, always to the US-PDT group’s benefit. Both protocols lowered the levels of IL-6 and TNF-a. The US-PDT group showed a significant decrease in IL-6 in week three and for TNF-a in week six. The amount of *P. intermedia* and *P. gingivalis* in the plaque samples was much lower in both follow-up periods in the case of the adjunct PDT group [31].

Malik et al. (2020) also compared the efficacy of aPDT in combination with US to US alone, but against oral yeast. At the beginning of the study and 6 months after the intervention, the gingival index was tested and unstimulated whole saliva (UWS) samples were collected. In the case of the GI, the results were comparable between the two groups. On the other hand, in the case of the yeast, there was a significant decrease in the amount of CFU/mL for the combined therapy, while the decrease for the US alone with respect to the baseline value was not statistically significant [32].

### 3.3. Comparison of Antimicrobial Photodynamic Therapy and Chlorhexidine Products

Panhoca et al. (2016) evaluated the efficacy of aPDT using curcumin, which was modified by adding sodium dodecyl sulfate (SDS) surfactant to improve activity against bacterial biofilm. The 24 patients treated with fixed orthodontic appliances were divided into four groups. The first group was exposed to light only, the second group underwent PDT with curcumin, the third group underwent PDT with modified PS containing SDS, and the last group of patients used a mouth rinse with chlorhexidine gluconate solution (CHX). The study used light sources with a wavelength of 450 nm ±+/− 10 nm. A statistically significant decrease in the number of bacteria was found in UWS samples for groups 2, 3, and 4. However, researchers found no significant differences between the PDT + SDS group and the CHX group [33].

Paschoal et al. (2015) compared the effects of aPDT with curcumin as PS with CHX varnish applied to teeth in the prophylaxis of white spot lesions and gingivitis. The mentioned therapies were applied four times at weekly intervals. Researchers compared PI and GBI at baseline one month after the intervention and three months after. In the case of the aPDT group, a light source with a wavelength of 450 nm, fluence of 96 J/cm^2^_,_ and a fluence rate of 165 mW/cm^2^ was used. After a one-month follow-up period, no significant changes in PI values were observed for all groups, but, at the 3-month follow-up period in the aPDT group, an increase in the aforementioned index was observed. The GBI reduction was present at the first follow-up visit and then returned to the baseline values [34].

### 3.4. Microbiological Analysis before and after the Application of Antimicrobial Photodynamic Therapy

Soares et al. (2019) assessed the antimicrobial activity of aPDT using a mixture of toluidine blue and methylene blue. They took plaque samples from patients before treatment, after rinsing the mouth with PS, and then after photoactivation. They found a statistically significant decrease in the amount of bacteria after the application of aPDT [35].

### 3.5. The Influence of Antimicrobial Photodynamic Therapy on Tongue Hygiene

Unpleasant mouth odor can be a problem for patients when there is insufficient tongue hygiene. Alshahrani et al. (2020) compared the use of tongue scraper (TS) and PDT to reduce halitosis. The study included 45 patients divided into three groups. The first group was subject to PDT with MB at a concentration of 0.005%, the second group used TS, and the last group used combination therapy with both agents. The researchers assessed the concentration of H_2_S using the Oral Chroma device and took samples from the tongue for microbiological evaluation. They noted a statistically significant decrease in the H_2_S level in all groups after two weeks of observation, with the most significant one for the PDT-TS group. In the microbiological evaluation, the same group showed a statistically significant decrease in the number of bacteria on the back of a tongue [36].

### 3.6. Effect of Photodynamic Therapy on Tooth Movement

El Shehawy et al. (2020) attempted to use PDT as a method of accelerating tooth movement. Patients reported weekly follow-up visits where the researchers took impressions for plaster models. They then scanned each model to obtain 3D digital models. The study group underwent PDT using MB on days 0, 3, 7, and 14 of the first month and then again in the following two months. There were no differences in the rate of tooth alignment in the anterior segment of the mandible arch between the study and the control group [37].

## 4. Ex Vivo Studies

### 4.1. Modified Orthodontic Adhesives

Ahmadi et al. (2020) assessed the anti-biofilm effect of the modified orthodontic adhesive (MOA). In their research, they used curcumin (cur), which they enclosed in poly (lactic-*co*-glycolic acid) (PLGA) nanoparticles to improve its bioavailability. MOA was created by adding Cur-PLGA-NPs to the commercially available Transbond XT (3 M, Unitek, Monrovia, CA, USA) in the amount needed to create 3, 5, 7, and 10% wt. Due to the highest concentration of released Cur-PLGA-NPs and the highest value of shear bond strength, MOA with a content of 7% wt was selected for the study of antibacterial activity. Brackets bonded to enamel slabs were artificially aged to a value corresponding to 180 days. The samples were irradiated with light with a wavelength of 405 ± 5 nm and a fluence rate of 150 mW/cm^2^. On days 15, 30, 60, 90, and 120, a statistically significant decrease in the optical density of *S. mutans* biofilm for the test sample was found in the violet crystal assay. However, from day 150 onwards, *S. mutans* biofilm growth was increasing. It follows that the addition of Cur-PLGA-NPs to OA and its subsequent photoactivation shows an antimicrobial effect, but for a period not exceeding 5 months [38].

Algerban (2021) created MOAs by adding riboflavin (RF) or rose bengal (RB) to Transbond XT in an amount necessary to create a final concentration of 0.1% and 0.5%. In the degree of conversion (DC), the study showed a good level of monomer conversion for MOAs with 0.1% RB lub 0.1% RF, which was comparable to the control group. In the scanning electron microscope(SEM) image, a good connection between the bracket and MOAs was visible, and a faulty connection was only found for 0.5% RF. The light source was used for illumination in the UVA range (375 nm, 3 mW/cm^2^). SEM depicted a decreased growth of *S. mutans* for MOAs compared to the control group; the decrease correlated with an increase in PSs concentration. The results of *S. mutans* viability were confirmed in the MTT assay performed on the 1st and 30th day of the study. There were no statistically significant differences for the adhesive remnant index (ARI) for all five groups [39].

Pourhajibagher et al. (2019) assessed the antimicrobial efficacy of their MOA by adding synthesized cationic curcumin doped zinc oxide nanoparticles (cCur/ZnONPs). In the first stage of the research, they determined the percentage by weight with the best mechanical properties. SBS decreased with increasing amounts of cCur/ZnONPs; however, starting from 7.5 wt., no statistical significance was demonstrated. Therefore, this MOA was selected for microbiological studies. There were no significant differences in ARI between the different MOA concentrations and the Transbond XT control group. The discs with bonded brackets were artificially aged. Until day 90, there was no growth of *S. mutans* and *S. sobrinus* bacteria and in the case of *L. acidophilus* until day 60. In the following days, despite the observation of growth, the values were significantly lower than in the control group. On day 180, the values approached those observed for the control group. The metabolic activity of the mentioned bacteria was also reduced [40].

### 4.2. Effect of Photodynamic Therapy on the Bond Strength

Baeshen (2021) compared the different conditioning methods of lithium disilicate (LDS), including PDT, before bonding metal brackets. Taking into account ARI and SBS, they confirmed that etching with hydrofluoric acid followed by silane application is still the gold standard in LDS surface preparation. The use of PDT with MB resulted in a reduction in bonding strength [41].

Mirhashemi et al. (2021) assessed the effect of the applied aPDT before bonding the orthodontic bracket on the SBS value. They compared MB and indocyanine green (ICG). The highest SBS was recorded for the control group, while a statistically significant decrease in binding strength occurred for both aPDT protocols. There was no statistically significant difference in the decrease in SBS values for both PSs [42].

Kamran et al. (2021) assessed riboflavin-mediated PDT against *S. mutans* and *S. sanguinis* in the oral cavity compared to 0.2% chlorhexidine (CHX). The MTT test showed a significant decrease in viability of both bacteria compared to the dark and untreated group, but no significant difference in viability was found between the PDT and CHX groups. These results were confirmed by the images obtained with confocal laser microscopy [43].

### 4.3. Antimicrobial Photodynamic Therapy in the Decontamination of Instruments and Orthodontic Appliances

Xie et al. (2020) synthesized deoxyribonuclease (DNase) decorated gold nanoclusters (AuNCs) (DNase-AuNCs) and then assessed the activity against the biofilm formed on Invisalign aligners upon activation of near infra-red light) NIR. DNase has the ability to destroy the extracellular polymeric substance (EPS), which is a biofilm component, thanks to which AuNCs can get inside the bacterial cell and cause an effective photodynamic and photothermal reaction. There was an 80% and 75% reduction in biofilm weight for *S. aureus* and *P. aeruginosa*, respectively. The bacterial inhibition ratio was about 90%. For comparison, the weight of the biofilm in the case of using CHX decreased by 55%, while for 75% alcohol, the weight decreased by 30% [44].

Foggiato et al. (2018) assessed the usefulness of a photodynamic inactivation device in the decontamination of orthodontic instruments. They developed a polypropylene case, lined with aluminum foil and containing LEDs emitting light with a wavelength of 660 nm. Previously autoclaved instruments were contaminated with a suspension of gram-positive bacteria Staphylococcus aureus and Streptococcus mutans or gram-negative *Escherichia coli*. The instruments were then incubated for 20 min in MB at a concentration of 100 µM/L and subsequently irradiated with fluence 26 J/cm² for 20 min. The researchers showed a statistically significant decrease in the CFU for the described method compared to the positive control [45].

Lacerda Rangel Esper et al. (2019) investigated the efficacy of hematoporphyrin IX (H) and modified hematoporphyrin IX (MH) exposed to blue light against planktonic bacteria and *S. mutans* biofilm. They found that both PSs were effective against the planktonic bacteria. In the case of the biolfilm, H proved ineffective for both metal and ceramic brackets. However, for MH, a significant difference in the amount of bacteria was shown, with a decrease of 44% and 53% in the amount of bacteria for metal and ceramic brackets, respectively [46].

## 5. Conclusions

There is a growing interest among researchers in the possibility of using PDT in orthodontics, which is confirmed by the fact that the vast majority of studies included in the above literature review are not older than two years. Two main groups of studies can be found in the literature, clinical studies on the use of aPDT in orthodontically treated patients and preclinical studies on the use of MOAs by adding to them PSs.

In clinical trials, MB is the most frequently used PS, followed by curcumin. The effectiveness of aPDT is mainly compared to US as a single therapy or as an adjunct to US. In their conclusions, the researchers most often emphasize the effectiveness of aPDT in reducing microbial levels in patients treated with fixed appliances and the possibility of using it as an alternative to routine procedures aimed at maintaining a healthy periodontium. The attempt to use PDT as a method to accelerate tooth movement did not bring satisfactory results.

However, these modifications do not reduce the bond strength of the enamel surface. A reduction in shear bond strength could result in more frequent failures of the detachment of orthodontic brackets and possible extension of treatment time, which could adversely affect oral health. PDT using MOAs presents encouraging results against cariogenic bacteria, but the time of effective antimicrobial activity is limited.

PDT can reduce bond strength, suggesting that this procedure should not be performed immediately prior to bonding orthodontic brackets.

Promising results in the use of PDT and aPDT in various fields of medicine have also increased interest in this method in the field of orthodontics. Al-Shammery et al., in their review, stated the need for further research on the use of aPDT to prove the validity of its use in orthodontics [47]. The authors of the current review also suggest further research in this direction.

Before applying a new method, the basic principles must not be forgotten. Invariably, proper hygiene and patient motivation are required to maintain good oral health. In addition, unsatisfactory oral hygiene is a contraindication to the initiation of orthodontic treatment; therefore, in such cases, it should be postponed until proper hygiene is achieved.

## Figures and Tables

**Table 1:** Photodynamic therapy in clinical trials.

Total Number of Patients	Study Design	Mean Age	Photosensitizer	light Parameters	Study Groups	Investigative Parameter	Authors
20	RCT	14.6 ± 1.6	Methylene blue 0.005%	670 nm, 67.06 J/cm^2^, 6.05 W/cm^2^	Group 1: aPDT Group 2: US alone	PD, PI, GI, Microbiological analysis, GCF cytokines assessment	Abellan et al. [25]
20	RCT	Group 1: 15.0 ± 1.8 Group 2: 14.2 ± 1.3	Methylene blue 0.005%	670 nm, 200 mW	Group 1: aPDT Group 2: US alone	FMPS, FMBS, PD, ICDAS, Microbiological analysis	Gómez et al. [26]
22	RCT	Group 1: 17.8 ± 0.7 Group 2: 17.3 ± 0.9	Methylene blue 0.0005%	670 nm, 22 J/cm^2^, 150 mW	Group 1: US Group 2: US + aPDT	PD, PScore, BoP, Microbiological analysis	Al Nazeh et al. [27]
45	RCT	Group 1: 14.7 ± 0.8 Group 2: 16.2 ± 0.9 Group 3: 15.8 ± 0.7	Methylene blue 0.0005%	670 nm, 22 J/cm^2^, 1.1 W/cm^2^	Group 1: US + aPDT, Group 2: US + PBM Group 3: US alone	PScore, BoP PD, Microbiological analysisGCF cytokines assessment	Alqerban [28]
26	CT	Group 1: 16.3 ± 0.9 Group 2: 16.9 ± 1.0	Methylene blue 0.0005%	670 nm, 22 J/cm^2^, 150 mW	Gruoup 1: FMPD Group 2: FMPD + aPDT	PScore, BoP, PD, HI, Microbiological analysis, GCF cytokines assessment	Alshahrani et al. [29]
30	RCT	Group 1: 16.1 ± 1.4 Group 2: 15.9 ± 1.3	Methylene blue 0.005%	670 nm, 22 J/cm^2^, 150 mW	Group 1: US + aPDT Group 2: US alone	PScore, BOP, PD, VAS, Microbiological analysis, GCF cytokines assessment	Baeshen et al. [30]
50	CT	Group 1: 15.4 ± 0.9 Group 2: 14.2 ± 0.7	Methylene blue 0.0005%	670 nm, 22 J/cm^2^, 150 mW	Group 1: US + aPDT Group 2: US alone	PD, BoP, PScore, Microbiological analysis, GCF cytokines assessment	Kamran [31]
36	RCT	Group 1: 16.6 ± 0.5 years Group 2: 16.8 ± 0.4 years	Methylene blue 400 μg/mL	660 nm, 0.0125 J/cm², 150 mW	Group 1: US + aPDT Group 2: US alone	GI, Oral yeasts analysis	Malik et al. [32]
24	RCT	N/A 18–50 years	Curcumin 1 g/L with 0.1% of SDS	450 ± 10 nm extra-oral irradiation: 14 J/cm^2^, 200 mW, intra-oral irradiation 85 J/cm^2^, 1200 mW,	Group 1: Light only Group 2: PDT group Group 3: PDT + surfactant Group 4: CHX group	Microbiological analysis	Panhoca et al. [33]
55	RCT	N/A 13–18 years	Curcumin 1.5 mg/mL	450 ± 20 nm, 96 J/cm2, 165 mW/cm^2^	Group 1: 2% CHX varnish Group 2: placebo varnish Group 3: aPDT	PI, GI	Paschoal et al. [34]
21	Cross-over clinical study	N/A	Methylene Blue + Toluidine Blue, 1:1, 12.5μg/mL	640 ± 5 nm, 30 J/cm^2^	Group 1: control Group 2: PS only Group 3: aPDT	Microbiological analysis	Soares et al. [35]
45	RCT	Group 1: 15.4 (±1.48) Group 2: 13.8 (±0.91) Group 3: 14.2 (±1.17)	Methylene blue of 0.005%	660 nm, 317.43 J/cm^2^, 100 mW,	Group 1: aPDT Group 2: tongue scrappers Group 3: tongue scrappers + aPDT	Breath analysis, Microbiological analysis	Alshahrani et al. [36]
30	RCT	19.23 ± 3.08	Methylene blue	635 nm, 6.5 J/cm^2^, 20 mW	Group 1: PDT Group 2: control	LII	El Shehawy [37]

Shortcuts: PD—pocket depth, PI—plaque index, GI—gingival index, GCF—gingival crevicular fluid, FMPS—full mouth plaque score, FMBS—full mouth bleeding score, ICDAS—International Caries Detection and Assessment System, PScore—plaque score, BoP—bleeding on probing, HI—hyperplastic index, RCT—randomized controlled trial.

**Table 2:** Photodynamic therapy in ex vivo research.

Photosensitizer	Light Parameters	Study Groups	Investigative Parameter	Author
Cur-PLGA-NPs	405 ± 5 nm, 150 mW/cm^2^	Transbond XT supplemented with 0, 3, 5, 7, and 10% wt. Cur-PLGA-NPs	ARI, SBS, Microbiological analysis	Ahmadi et al. [38]
0.1, 0.5 wt.% Rose Bengal or Riboflavin	375 nm, 3 mW/cm^2^	Group 1: Transbond XT, Group 2: 0.1% RB–PDT adhesive, Group 3: 0.1% RF–PDT adhesive, Group 4: 0.5% RB–PDT adhesive, Group 5: 0.5% RF–PDT adhesive	DC, MTT assay, ARI, SEM	Alqerban [39]
cCur/ZnONPs	435 ± 20 nm, 300–420 J/cm^2^ 1000–1400 mW/cm^2^	Transbond XT supplemented with 0, 1.2, 2.5, 5, 7.5, and 10% wt. cCur/ZnONPs	SBS, ARI, Crystal violet assay, XTT assay, DAD, biofilm formation inhibition,	Pourhajibagher et al. [40]
Methylene Blue 100 mg/L	810 nm	Group 1: Er-YAG laser + silane Group 2: PDT + silane, Group 3: H F + S Group 4: H F + Ultrasonic Bath + S, Group 5: sand blasting, Group 6: self-etch glass ceramic primer Group 7: Er,Cr:YSGG laser + silane	SBS, ARI	Baeshen [41]
methylene blue indocyanine green	660 nm, 14.4 J/cm^2^, 150 mW 808 nm, 24 J/cm^2^, 250 mW,	Group 1: control Group2: aPDT MB, Group 3: aPDT ICG	SBS, ARI, SEM	Mirhashemi et al. [42]
Riboflavin 0.5%,	450 ± 65 nm, 95 J/cm^2^,	Group 1: riboflavin + LED irradiation; Group 2: riboflavin alone; Group 3: 0.2% chlorhexidine gluconate Group 4: not submitted to any treatment.	MTT assay, confocal laser microscopy, microbiological analysis	Kamran et al. [43]
DNase-AuNCs 200 μg/mL	808 nm, 2 W/cm^2^	Group 1: DNase-only, Group 2: BSA-AuNCs-only Group 3: DNase-AuNCs-only, Group 4: DNase-AuNCs plus NIR Group 5: control	Crystal violet staining, SEM	Xie et al. [44]
Methylene blue 100 μmol/L	660 nm 26 J/cm^2^	Group 1: *S. aureus* Group 2: *S. mutans* Group 3: *E. coli*	Microbiological analysis	Foggiato et al. [45]
Hematoporphyrin IX and modified hematoporphyrin IX 10 μmol/L	420–480 nm, 75 J/cm^2^, 1250 mW/cm^2^	Group 1: PDT hematoporphyrin IX Group 2: Hematoporphyrin IX only Group 3: PDT with modified hematoporphyrin IX Group 4: Modified hematoporphyrin IX only Group 5: LED irradiation only Group 6: No LED irradiation or photosensitizer	Microbiological analysis	Lacerda Rangel Esper et al. [46]

Shortcuts: DC—Degree of Conversion, ARI—Adhesive Remnant Index, SEM—scanning electron microscope, SBS—shear bond strength, DAD—disc agar diffusion.

## Data Availability

Department of Orthodontics, Faculty of Medical Sciences in Zabrze, Medical University of Silesia, 40-055 Katowice, Poland.
